# Globaltest and GOEAST: two different approaches for Gene Ontology analysis

**DOI:** 10.1186/1753-6561-3-S4-S10

**Published:** 2009-07-16

**Authors:** Ina Hulsegge, Arun Kommadath, Mari A Smits

**Affiliations:** 1 Animal Breeding and Genomics Centre, Animal Sciences Group Wageningen UR, P.O. Box 65, 8200 AB Lelystad, The Netherlands

## Abstract

**Background:**

Gene set analysis is a commonly used method for analysing microarray data by considering groups of functionally related genes instead of individual genes. Here we present the use of two gene set analysis approaches: Globaltest and GOEAST.

Globaltest is a method for testing whether sets of genes are significantly associated with a variable of interest. GOEAST is a freely accessible web-based tool to test GO term enrichment within given gene sets. The two approaches were applied in the analysis of gene lists obtained from three different contrasts in a microarray experiment conducted to study the host reactions in broilers following *Eimeria *infection.

**Results:**

The Globaltest identified significantly associated gene sets in one of the three contrasts made in the microarray experiment whereas the functional analysis of the differentially expressed genes using GOEAST revealed enriched GO terms in all three contrasts.

**Conclusion:**

Globaltest and GOEAST gave different results, probably due to the different algorithms and the different criteria used for evaluating the significance of GO terms.

## Background

Several methods have recently been developed for gene set analysis of microarray data [1,2]. These methods evaluate differential gene expression patterns of groups of functionally related genes instead of individual genes. The aim is to discover gene sets whose expression patterns are associated with phenotypes of interest. Genes can be grouped together into gene sets, for example, based on function (Kyoto Encyclopedia of Genes and Genomes (KEGG), Gene Ontology (GO) [3]) or location (chromosome, cytoband). In this paper we present the results obtained with two different gene set analysis approaches: Globaltest [4] and Gene Ontology Enrichment Analysis Software Toolkit (GOEAST) [5]. Globaltest is a method for testing whether sets of genes are significantly associated with a variable of interest. The method is based on a prediction model for predicting a response variable from the gene expression measurements of a set of genes. The null hypothesis tested is that expression profile of the genes in the gene set is not associated with the response variable. GOEAST is a freely accessible web-based tool to test GO term enrichment within given gene sets. It supports the analysis of data from common commercial microarray platforms and even customized arrays if the probe annotation file in the required format is provided.

These approaches were applied in the analysis of gene lists obtained from three different contrasts in a microarray experiment conducted to study the host reactions in broilers following *Eimeria *infection.

## Methods

### Globaltest

The Globaltest allows different kinds of variables to be tested, based on which it determines the correct model (logistic, linear or survival).

The Globaltest calculates the p-value using different methods, the most important ones being permutations and the asymptotic distribution. Here the asymptotic distribution was used. All p-values were corrected for multiple testing using Benjamini and Hochberg's False Discovery Rate (FDR) [6]. GO terms were considered significant if the p-value after correcting for multiple testing, was below 0.05. The influence of individual genes in a GO term was evaluated using z-score calculated in Globaltest. Genes with z-scores that are greater than 2 were considered significant contributors to the GO term. GO terms which matched only one gene were excluded from the analysis.

The Globaltest package also offers plots to visualize the effects of different genes and different samples on the test result: 1. Sample plot: how good a sample fits to its phenotype, 2. Checkerboard: correlation between samples, and 3. Gene plot: Influence of individual genes to test statistics.

R version 2.8.0 was used to run the Globaltest package (version 4.12.0).

#### Availability

Globaltest: 

R: 

### GOEAST

For GOEAST all GO terms with less than 5 probes associated with it on the array are discarded from the test because the statistical analysis would not be appropriate then.

The Fisher's exact test available in GOEAST was used separately on the 2-fold upregulated and downregulated gene lists for each of the three contrasts. The p-values were adjusted using Benjamini-Yekutieli method [7] with cutoff for FDR control set at 0.1. The Benjamini-Yekutieli method is more suitable for positively related multiple tests as is the case for enriched GO terms within gene lists [5]. To reduce the FDRs caused by over-representation of neighbouring GO terms due to their hierarchical dependency, Adrian Alexa's improved weighted scoring algorithm [8] which is implemented in GOEAST was used.

The results from GOEAST analysis are presented in 3 ways: an HTML table providing detailed information of enriched GO terms and their associated genes; a plain-text file of enriched GO terms; and separate graphical output files showing the hierarchical relationships of enriched GO terms in the 3 GO categories.

Besides the Fisher's exact test, GOEAST also supports hypergeometric test and χ^2^-test as well as other methods for multiple testing correction (Hochberg, Bonferroni, Hommel).

#### Availability



## Results

### Globaltest

The Globaltest takes into account the entire raw expression data. The overall gene expression profile for the three contrasts (MM8-PM8, MM8-MA8 and MM8-MM24) was significantly associated (p < 0.05) with their outcomes, the p-values using the asymptotic method being 0.006, 0.032 and 0.021 respectively. This shows that the overall gene expression pattern of MM8 chicken differs significantly from that of PM8, MA8 and MM24 chicken. Therefore there is a potential in predicting infection from gene expression data.

**Figure 1:**
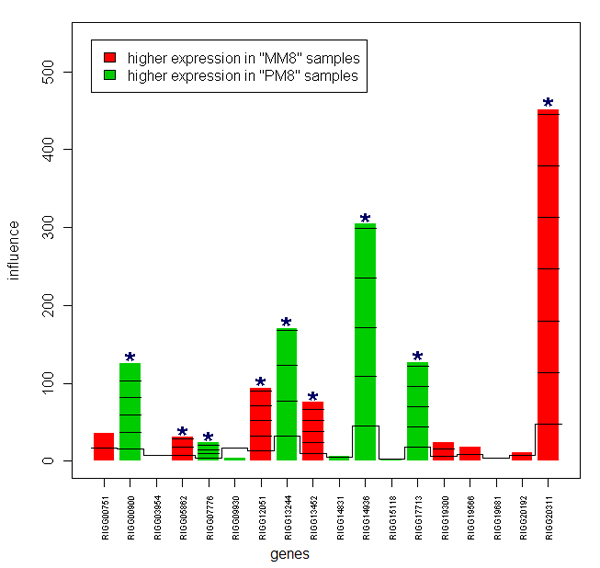
**Geneplot of the GO term "ruffle"**. The gene plot shows a bar and a reference line for each gene tested. The reference line reflects the expected influence if the gene was not associated with the GO term "ruffle". In case the height of the bar exceeds the reference line, the gene significantly influences the GO term "ruffle". Marks indicate the standard deviations by which the bar exceeds the reference line. The bars are coloured to indicate a higher expression in PM8 compared to MM8 (green) or a higher expression in MM8 compared to PM8 (red). * Genes clearly above the reference.

GO terms (biological process, molecular function, cellular component) were used for gene set analysis. After correction for multiple testing, no significant gene sets (GO terms) were found in MM8-MA8 and MM8-MM24 contrasts. However, in the MM8-PM8 contrast, 527, 331 and 180 out of a total of 1679, 838 and 336 GO-terms, were found to be significant (p < 0.05) for biological process, molecular function and cellular component, respectively. The five most significant terms for each GO category are listed in Table 1. The influence of individual genes on the results for the GO term "ruffle" is shown in Figure 1. Nine genes were clearly above the reference line and nine genes did not show an effect.

**Table 1:** Top 5 GO terms in contrast MM8-PM8 identified by Globaltest

GO term ID	GO term description	Number of genes in GO term^a^	Number of genes affected^b^	p-value	FDR adjusted p-value^c^
*Biological Process*					
GO:0051017	actin filament bundle formation	6	2	0.002	0.047
GO:0006996	organelle organization and biogenesis	3	2	0.002	0.047
GO:0015816	glycine transport	2	1	0.003	0.047
GO:0016042	lipid catabolic process	7	6	0.003	0.047
GO:0009113	purine base biosynthetic proces	4	2	0.003	0.047
*Molecular function*					
GO:0019976	interleukin-2 binding	2	2	0.002	0.040
GO:0015187	glycine transmembrane transporter activity	2	1	0.003	0.040
GO:0031013	troponin I binding	2	1	0.003	0.040
GO:0003847	1-alkyl-2-acetylglycerophosphocholine....	2	2	0.003	0.040
GO:0004438	phosphatidylinositol-3-phosphatase activity	2	2	0.003	0.040
*Cellular component*					
GO:0001726	ruffle	18	9	0.003	0.034
GO:0005719	nuclear euchromatin	2	1	0.003	0.034
GO:0005884	actin filament	12	6	0.003	0.034
GO:0000307	cyclin-dependent protein kinase...	5	3	0.004	0.034
GO:0016529	sarcoplasmic reticulum	4	3	0.004	0.034

^a ^Number of genes within each GO term.

^b ^Genes differentially expressed with z-score > 2.0

^c ^The p-values were adjusted for multiple hypotheses testing with Benjamini and Hochberg to control the false discovery rate (FDR).

### GOEAST

In GOEAST, only differentially expressed genes over 2 fold level within the gene lists from each of the three contrasts were taken into account. The analysis for enriched GO terms by the Fisher's exact test revealed a large number of enriched GO terms at 0.1 FDR level for all three contrasts (Table 2). GOEAST identified 34, 12 and 39 enriched GO terms at FDR 0.1 level for the up regulated genes in the three contrasts MM8-PM8, MM8-MA8 and MM8-MM24 respectively, whereas for the down regulated genes, 35, 31 and 57 enriched GO terms were identified in the three contrasts respectively. A relatively high number of enriched GO terms were found within the small number of differentially expressed genes in the contrast MM8-MA8 (Table 2). This is probably due to the fact that many of the enriched GO terms consisted of just 1 or 2 well annotated genes. For example, several GO terms consisted of the gene *TICAM1 *alone. The top 5 GO terms within each GO category that were significantly enriched among differentially expressed genes for the 3 contrasts are presented in Additional file 1. The highest number of enriched GO terms were found for the GO category biological process.

**Table 2:** Summary of results of Fisher's exact test in GOEAST for the 3 contrasts

Contrast	Number of significantly expressed genes (>2 fold up/down regulation)		Number of genes with GO annotation	Number of enriched GO terms (adjusted p-value<0.1)
MM8-PM8	up	659	303	34
	down	647	216	35
MM8-MA8	up	22	9	12
	down	57	13	31
MM8-MM24	up	131	58	39
	down	515	146	57

## Discussion

In this study, two different approaches for gene set analysis were used to analyse three contrasts made in a microarray experiment. The Globaltest is a method for testing whether sets of genes are significantly associated with a variable of interest. GOEAST, a web based software, tests for enriched GO terms in specified gene sets.

The Globaltest is a direct gene set testing method and does not start from a list of differential expressed genes, but from the raw expression data. An advantage of Globaltest compared to GOEAST is its ability to identify GO terms with genes that have limited changes in gene expression. With Globaltest, enriched GO terms can be found because only a few genes are highly differentially expressed or because many genes are only slightly differentially expressed. This may help to distinguish the key player genes of the affected GO term. The identification of genes contributing more or less to particular biological processes and molecular functions may be of great help in guiding further investigation of the pathways.

For Globaltest, given the small sample size (10 microarrays) a permutation distribution could not generate a unique p-value and therefore the asymptotic distribution was used. Although the asymptotic distribution is correct for large sample sizes, it also gives a good indication for small sample sizes [4].

From GOEAST results, it was noted that several enriched GO terms were associated with only 1 or a few genes in the tested gene lists. Though the terms still appear to be statistically significant, their biological relevance should be carefully looked into.

For example, 3 among the top 5 GO 'biological process' terms enriched in the list of down regulated genes of the contrast MM8-MA8 had one and the same gene, *TICAM1*, annotated to that term. However, these terms may still be biologically relevant since the *TICAM1 *gene is known to be involved in innate immunity against invading pathogens and therefore important in the context of the experiment that generated the gene lists.

We found different results for the two methods probably due to the different algorithms used and also the different criteria used for evaluating the significance of GO terms. Different results achieved by different gene set analysis methods were previously reported by other authors [2,9].

## Conclusion

The Globaltest and GOEAST gave different results, probably due to the different algorithms and also the different criteria used for evaluating the significance of GO terms. This confirms that different gene set analysis methods perform differently and that they do not necessarily lead to the same biological conclusions. A pitfall in interpretation of the results presented here is the lack of sufficient annotation of the probes used in this microarray experiment.

## List of abbreviations used

FDR: False Discovery Rate; GO: Gene Ontology; GOEAST: Gene Ontology Enrichment Analysis Software Toolkit; KEGG: Kyoto Encyclopedia of Genes and Genomes

## Competing interests

The authors declare that they have no competing interests.

## Authors' contributions

The three authors contributed equally to this paper.

## Supplementary Material

Additional file 1 **Top 5 GO terms identified by GOEAST for each microarray contrast**. This file presents the top 5 GO terms within each GO category identified by Fisher's exact test in GOEAST to be significantly enriched among the >2 fold differentially expressed genes for the 3 contrasts: MM8-PM8, MM8-MA8 and MM8-MM24 Click here for file
