# Supplementary material for: Globaltest and GOEAST: two different approaches for Gene Ontology analysis
Source: BMC Proc. 2009 Jul 16;3(Suppl 4):S10. doi: 10.1186/1753-6561-3-S4-S10 (PMC2712740; doi:10.1186/1753-6561-3-S4-S10)
Supplement: Additional file 1 — Top 5 GO terms identified by GOEAST for each microarray contrast. This file presents the top 5 GO terms within each GO category identified by Fisher's exact test in GOEAST to be significantly enriched among the >2 fold differentially expressed genes for the 3 contrasts: MM8-PM8, MM8-MA8 and MM8-MM24 [file 1753-6561-3-S4-S10-S1.doc]

## The top 5 significantly enriched GO terms in contrast MM8-PM8 identified by GOEAST

| **GO ID** | **GO Term** | **q** | **m** | **t** | **k** | **p-value** |
| --- | --- | --- | --- | --- | --- | --- |
| **UPREGULATED GENES** | | | | | | |
| *biological process* | | | | | | |
| GO:0006749 | glutathione metabolic process | 3 | 8 | 7099 | 303 | 0.003 |
| GO:0007005 | mitochondrion organization | 5 | 44 | 7099 | 303 | 0.010 |
| GO:0006921 | cell structure disassembly during apoptosis | 4 | 14 | 7099 | 303 | 0.013 |
| GO:0042110 | T cell activation | 4 | 35 | 7099 | 303 | 0.023 |
| GO:0001934 | positive regulation of protein amino acid phosphorylation | 3 | 16 | 7099 | 303 | 0.023 |
| *molecular function* | | | | | | |
| GO:0003723 | RNA binding | 14 | 138 | 7099 | 303 | 0.002 |
| GO:0017022 | myosin binding | 3 | 10 | 7099 | 303 | 0.007 |
| GO:0008009 | chemokine activity | 3 | 14 | 7099 | 303 | 0.019 |
| GO:0005545 | phosphatidylinositol binding | 2 | 6 | 7099 | 303 | 0.023 |
| GO:0015036 | disulfide oxidoreductase activity | 1 | 5 | 7099 | 303 | 0.023 |
| *cellular component* | | | | | | |
| GO:0000307 | cyclin-dependent protein kinase holoenzyme complex | 2 | 5 | 7099 | 303 | 0.017 |
| GO:0005657 | replication fork | 3 | 16 | 7099 | 303 | 0.029 |
| GO:0031674 | I band | 3 | 27 | 7099 | 303 | 0.043 |
| GO:0042101 | T cell receptor complex | 2 | 8 | 7099 | 303 | 0.043 |
| GO:0009897 | external side of plasma membrane | 5 | 47 | 7099 | 303 | 0.049 |
| **DOWNREGULATED GENES** | | | | | | |
| *biological process* | | | | | | |
| GO:0051004 | regulation of lipoprotein lipase activity | 2 | 6 | 7099 | 216 | 0.004 |
| GO:0030501 | positive regulation of bone mineralization | 2 | 5 | 7099 | 216 | 0.007 |
| GO:0000271 | polysaccharide biosynthetic process | 2 | 6 | 7099 | 216 | 0.015 |
| GO:0006641 | triacylglycerol metabolic process | 3 | 21 | 7099 | 216 | 0.019 |
| GO:0045765 | regulation of angiogenesis | 3 | 21 | 7099 | 216 | 0.019 |
| *molecular function* | | | | | | |
| GO:0030274 | LIM domain binding | 2 | 5 | 7099 | 216 | 0.008 |
| GO:0008201 | heparin binding | 4 | 29 | 7099 | 216 | 0.010 |
| GO:0005416 | cation:amino acid symporter activity | 2 | 6 | 7099 | 216 | 0.012 |
| GO:0030528 | transcription regulator activity | 24 | 487 | 7099 | 216 | 0.013 |
| GO:0005164 | tumor necrosis factor receptor binding | 2 | 7 | 7099 | 216 | 0.017 |
| *cellular component* | | | | | | |
| GO:0001726 | ruffle | 4 | 21 | 7099 | 216 | 0.003 |
| GO:0005925 | focal adhesion | 3 | 20 | 7099 | 216 | 0.021 |
| GO:0005681 | spliceosome | 3 | 20 | 7099 | 216 | 0.021 |
| GO:0005922 | connexon complex | 2 | 9 | 7099 | 216 | 0.028 |
| GO:0042627 | chylomicron | 2 | 9 | 7099 | 216 | 0.028 |

q: Number of probes associated with the listed GO ID (directly or indirectly) in the test dataset.

m: Number of probes associated with the listed GO ID (directly or indirectly) on the array.

k: Total number of probes in the test dataset.

t: Total number of probes on the array with GO annotation.

## The top 5 significantly enriched GO terms in contrast MM8-MA8 identified by GOEAST

| **GO ID** | **GO Term** | **q** | **m** | **t** | **k** | **p-value** |
| --- | --- | --- | --- | --- | --- | --- |
| **UPREGULATED GENES** | | | | | | |
| *biological process* | | | | | | |
| GO:0006098 | pentose-phosphate shunt | 1 | 8 | 7099 | 9 | 0.008 |
| GO:0006094 | gluconeogenesis | 1 | 8 | 7099 | 9 | 0.010 |
| GO:0006044 | N-acetylglucosamine metabolic process | 1 | 10 | 7099 | 9 | 0.010 |
| GO:0006096 | glycolysis | 1 | 21 | 7099 | 9 | 0.022 |
| GO:0006633 | fatty acid biosynthetic process | 1 | 21 | 7099 | 9 | 0.024 |
| *molecular function* | | | | | | |
| GO:0016861 | intramolecular oxidoreductase activity, interconverting aldoses and ketoses | 2 | 6 | 7099 | 9 | 0.000 |
| GO:0017137 | Rab GTPase binding | 1 | 13 | 7099 | 9 | 0.015 |
| GO:0003779 | actin binding | 1 | 98 | 7099 | 9 | 0.095 |
| *cellular component* | |  |  |  |  |  |
| GO:0008021 | synaptic vesicle | 1 | 27 | 7099 | 9 | 0.034 |
| **DOWNREGULATED GENES** | | | | | | |
| *biological process* | | | | | | |
| GO:0043123 | positive regulation of I-kappaB kinase/NF-kappaB cascade | 2 | 54 | 7099 | 13 | 0.008 |
| GO:0008090 | retrograde axon cargo transport | 1 | 5 | 7099 | 13 | 0.013 |
| GO:0032496 | response to lipopolysaccharide | 1 | 5 | 7099 | 13 | 0.013 |
| GO:0043330 | response to exogenous dsRNA | 1 | 5 | 7099 | 13 | 0.013 |
| GO:0045080 | positive regulation of chemokine biosynthetic process | 1 | 5 | 7099 | 13 | 0.013 |
| *molecular function* | |  |  |  |  |  |
| GO:0004623 | phospholipase A2 activity | 1 | 6 | 7099 | 13 | 0.012 |
| GO:0050681 | androgen receptor binding | 1 | 8 | 7099 | 13 | 0.016 |
| GO:0005525 | GTP binding | 2 | 113 | 7099 | 13 | 0.021 |
| GO:0004871 | signal transducer activity | 4 | 584 | 7099 | 13 | 0.025 |
| GO:0042054 | histone methyltransferase activity | 1 | 14 | 7099 | 13 | 0.028 |
| *cellular component* | |  |  |  |  |  |
| GO:0060053 | neurofilament cytoskeleton | 1 | 5 | 7099 | 13 | 0.013 |
| GO:0032473 | external side of mitochondrial outer membrane | 1 | 5 | 7099 | 13 | 0.013 |
| GO:0005721 | centromeric heterochromatin | 1 | 8 | 7099 | 13 | 0.020 |
| GO:0008305 | integrin complex | 1 | 13 | 7099 | 13 | 0.033 |

q: Number of probes associated with the listed GO ID (directly or indirectly) in the test dataset.

m: Number of probes associated with the listed GO ID (directly or indirectly) on the array.

k: Total number of probes in the test dataset.

t: Total number of probes on the array with GO annotation.

## The top 5 significantly enriched GO terms in contrast MM8-MM24 identified by GOEAST

| **GO ID** | **GO Term** | **q** | **m** | **t** | **k** | **p-value** |
| --- | --- | --- | --- | --- | --- | --- |
|  | | | | | | |
| **UPREGULATED GENES** | |  |  |  |  |  |
| *biological process* | | | | | | |
| GO:0007264 | small GTPase mediated signal transduction | 5 | 98 | 7099 | 58 | 0.003 |
| GO:0006298 | mismatch repair | 2 | 14 | 7099 | 58 | 0.005 |
| GO:0009113 | purine base biosynthetic process | 1 | 5 | 7099 | 58 | 0.037 |
| GO:0045910 | negative regulation of DNA recombination | 1 | 6 | 7099 | 58 | 0.037 |
| GO:0008340 | determination of adult life span | 1 | 5 | 7099 | 58 | 0.037 |
| *molecular function* | | | | | | |
| GO:0003682 | chromatin binding | 4 | 64 | 7099 | 58 | 0.002 |
| GO:0000287 | magnesium ion binding | 3 | 81 | 7099 | 58 | 0.028 |
| GO:0043531 | ADP binding | 1 | 6 | 7099 | 58 | 0.048 |
| GO:0005545 | phosphatidylinositol binding | 1 | 6 | 7099 | 58 | 0.048 |
| GO:0051059 | NF-kappaB binding | 1 | 6 | 7099 | 58 | 0.048 |
| *cellular component* | | | | | | |
| GO:0044454 | nuclear chromosome part | 3 | 45 | 7099 | 58 | 0.005 |
| GO:0005762 | mitochondrial large ribosomal subunit | 1 | 7 | 7099 | 58 | 0.052 |
| GO:0030426 | growth cone | 1 | 10 | 7099 | 58 | 0.074 |
| GO:0005747 | mitochondrial respiratory chain complex I | 1 | 11 | 7099 | 58 | 0.081 |
| GO:0031941 | filamentous actin | 1 | 13 | 7099 | 58 | 0.095 |
| **DOWNREGULATED GENES** | |  |  |  |  |  |
| *biological process* | | | | | | |
| GO:0007602 | phototransduction | 3 | 10 | 7099 | 146 | 0.001 |
| GO:0007606 | sensory perception of chemical stimulus | 3 | 18 | 7099 | 146 | 0.005 |
| GO:0018298 | protein-chromophore linkage | 2 | 6 | 7099 | 146 | 0.006 |
| GO:0001578 | microtubule bundle formation | 2 | 6 | 7099 | 146 | 0.006 |
| GO:0045086 | positive regulation of interleukin-2 biosynthetic process | 2 | 7 | 7099 | 146 | 0.008 |
| *molecular function* | | | | | | |
| GO:0030528 | transcription regulator activity | 20 | 487 | 7099 | 146 | 0.004 |
| GO:0008138 | protein tyrosine/serine/threonine phosphatase activity | 2 | 10 | 7099 | 146 | 0.017 |
| GO:0030674 | protein binding, bridging | 2 | 12 | 7099 | 146 | 0.025 |
| GO:0050750 | Low-density lipoprotein receptor binding | 2 | 12 | 7099 | 146 | 0.025 |
| GO:0003682 | chromatin binding | 4 | 64 | 7099 | 146 | 0.044 |
| *cellular component* | | | | | | |
| GO:0001725 | stress fiber | 3 | 13 | 7099 | 146 | 0.002 |
| GO:0001739 | Sex chromatin | 2 | 8 | 7099 | 146 | 0.012 |
| GO:0000792 | heterochromatin | 4 | 25 | 7099 | 146 | 0.050 |
| GO:0005681 | spliceosome | 2 | 20 | 7099 | 146 | 0.069 |
| GO:0005925 | focal adhesion | 2 | 20 | 7099 | 146 | 0.069 |

q: Number of probes associated with the listed GO ID (directly or indirectly) in the test dataset.

m: Number of probes associated with the listed GO ID (directly or indirectly) on the array.

k: Total number of probes in the test dataset.

t: Total number of probes on the array with GO annotation.
